# Novel Insights into How Overnutrition Disrupts the Hypothalamic Actions of Leptin

**DOI:** 10.3389/fendo.2018.00089

**Published:** 2018-03-26

**Authors:** Stefanie Fruhwürth, Heike Vogel, Annette Schürmann, Kevin Jon Williams

**Affiliations:** ^1^Department of Molecular and Clinical Medicine, Wallenberg Laboratory, Sahlgrenska University Hospital, Gothenburg, Sweden; ^2^Department of Experimental Diabetology, German Institute of Human Nutrition Potsdam-Rehbruecke, Nuthetal, Germany; ^3^German Center for Diabetes Research (DZD), München-Neuherberg, Germany; ^4^Department of Medicine, Section of Endocrinology, Diabetes, and Metabolism, Lewis Katz School of Medicine, Temple University, Philadelphia, PA, United States

**Keywords:** leptin, hypothalamus, obesity, energy balance, signaling

## Abstract

Obesity has become a worldwide health problem, but we still do not understand the molecular mechanisms that contribute to overeating and low expenditure of energy. Leptin has emerged as a major regulator of energy balance through its actions in the hypothalamus. Importantly, obese people exhibit high circulating levels of leptin, yet the hypothalamus no longer responds normally to this hormone to suppress appetite or to increase energy expenditure. Several well-known hypotheses have been proposed to explain impaired central responsiveness to the effects of leptin in obesity, including defective transit across the blood–brain barrier at the arcuate nucleus, hypothalamic endoplasmic reticulum stress, maladaptive sterile inflammation in the hypothalamus, and overexpression of molecules that may inhibit leptin signaling. We also discuss a new explanation that is based on our group’s recent discovery of a signaling pathway that we named “NSAPP” after its five main protein components. The NSAPP pathway consists of an oxide transport chain that causes a transient, targeted burst in intracellular hydrogen peroxide (H_2_O_2_) to inactivate redox-sensitive members of the protein tyrosine phosphatase gene family. The NSAPP oxide transport chain is required for full activation of canonical leptin signaling in neurons but fails to function normally in states of overnutrition. Remarkably, leptin and insulin both require the NSAPP oxide transport chain, suggesting that a defect in this pathway could explain simultaneous resistance to the appetite-suppressing effects of both hormones in obesity.

## Introduction

In just the past few decades, obesity has become a worldwide health problem. The underlying cause is excessive food intake and a sedentary lifestyle, resulting in a chronic positive energy imbalance. The maintenance of a healthy energy balance is essential for the prevention of obesity. Successful strategies to achieve sustained weight loss to reverse obesity must address appetite and food intake as well as energy expenditure. Why has keeping a healthy energy balance become so difficult recently?

A major regulator of appetite and hence food intake is the adipocyte-derived hormone leptin (1, 2). Because blood levels of leptin rise chronically in proportion to body fat mass (3), this hormone indicates caloric prosperity. In addition, blood concentrations of leptin concentrations rise acutely after a carbohydrate-containing meal, apparently stimulated by insulin (4, 5). Accordingly, leptin enters the central nervous system (CNS) where it provokes specific neuronal signals in the hypothalamus that mediate leptin’s appetite-suppressing effects (6, 7). In addition to these direct homeostatic actions in the hypothalamus, leptin has gained recognition as a modulator of neural circuits governing motivation and reward (8–10). Leptin acts *via* the mesolimbic dopaminergic “reward system” to suppress the motivational drive to seek and consume food (11, 12). Leptin also triggers responses in the CNS that can increase energy expenditure, e.g., *via* activation of non-shivering thermogenesis in brown adipose tissue (BAT) and the induction of BAT-like thermogenesis in white adipose tissue (WAT). This latter process is known colloquially as “browning” and is discussed in more detail below.

Obese individuals typically exhibit high circulating levels of leptin because overall fat mass is increased, yet the hypothalamus no longer responds normally to leptin to suppress appetite (13–15). The exact molecular mechanisms responsible for the poor response to leptin in the brain remain unknown. Here, we review several well-known hypotheses that have been proposed to explain impaired central responsiveness to the appetite-suppressing effects of leptin in obesity. We also discuss a new explanation that is based on our group’s recent discovery of a signaling pathway—the NSAPP oxide transport chain—that is required for full activation of canonical leptin signaling in neurons but fails to function normally in states of overnutrition.

## Central Regulation of Energy Balance—Normal Canonical Effects of Leptin

The first studies showing that a hormone from the circulation regulates feeding centrally took advantage of a pair of spontaneous mutant mice with severe heritable hyperphagia and obesity (16). One of these obese mice, *ob/ob*, was later used to identify the leptin gene, where the obesogenic mutation resides (2). The other obese mouse, *db/db*, carries two copies of a defective allele in the gene encoding the long form of the leptin receptor, LepRb (*Lepr^db/db^*). The *db/db* mouse is a key animal model of overnutrition, obesity, and type 2 diabetes mellitus. Restoration of CNS expression of functional LepRb in *db/db* mice is sufficient to normalize food intake and reverse their obesity (17).

The primary CNS site involved in the regulation of appetite by leptin is the arcuate nucleus of the hypothalamus (ARC). Within the ARC, leptin acts on LepRb, to inhibit neurons that express the orexigenic (appetite-stimulating) neuropeptide agouti-related peptide (AgRP), while simultaneously stimulating nearby neurons that express the anorexigenic (appetite-suppressing) neuropeptide proopiomelanocortin (POMC). Both of these actions work together to reduce food intake (6, 7).

**Figure 1:**
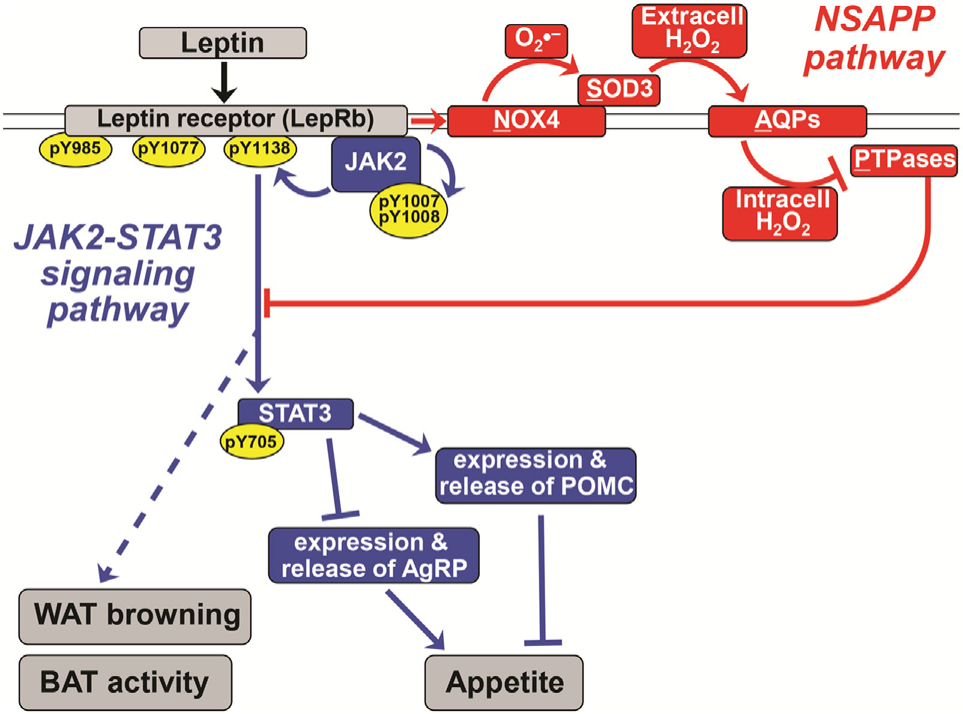
A novel pathway essential for canonical leptin signaling in hypothalamic neurons. Leptin activates signaling pathways to control the expression of appetite-regulating neuropeptides [proopiomelanocortin (POMC) and agouti-related peptide (AgRP)] in hypothalamic neurons. The canonical Janus kinase 2 (JAK2)–signal transducer and activator of transcription 3 (STAT3) signaling pathway is shown in blue. In red is the newly discovered NSAPP pathway that we showed to be essential for producing an intracellular burst of H_2_O_2_ that inhibits key protein tyrosine phosphatases (PTPases), thereby allowing canonical leptin signaling to propagate. Normal leptin signaling is required to suppress appetite and to stimulate white adipose tissue (WAT) browning and brown adipose tissue (BAT) thermogenic activity. Pointed arrowheads indicate stimulation of the immediately downstream molecule or process; flat arrowheads indicate inhibition. Specific phosphorylation sites are indicated within yellow ovals. The double line indicates the plasma membrane; all molecules above it are extracellular, and all named molecules below it in the schematic are intracellular. The leptin receptor (LepRb), NADPH oxidase-4 (NOX4), and aquaporins are transmembrane molecules. Solid lines represent experimentally demonstrated pathways; dotted lines are less well-characterized or putative. Adapted from Ref. (21–23).

Although leptin drives AgRP and POMC expression in opposite directions, the hormone acts in those two types of ARC neurons primarily *via* the same signaling cascades. Binding of leptin to the LepRb, which has no intrinsic kinase activity, causes the receptor to recruit intracellular Janus kinase 2 (JAK2). The recruitment of JAK2 in turn activates diverse signal transduction cascades through autophosphorylation and phosphorylation of LepRb and signal transducer and activator of transcription 3 (STAT3) (blue in Figure 1). Phosphorylation of STAT3 activates it, to silence the *AgRP* gene and drive expression of the *Pomc* gene. Leptin–LepRb binding also activates intracellular phosphoinositide-3 kinase (PI3K) (18) and causes phosphorylation and hence nuclear exclusion of the transcription factor FOXO1 (19). Consistent with this model, unphosphorylated FOXO1 and phosphorylated STAT3 appear to act on the *Agrp* and *Pomc* promoters through squelching, meaning that the two proteins compete for binding to overlapping sites within the two promoters (19). Both JAK2–STAT3 and PI3K have been implicated in leptin’s anorexigenic effect [reviewed in Ref. (20, 21)].

Regarding energy expenditure, leptin activates additional pathways in the brain that trigger efferent outflow to adipose tissue to alter its metabolism. Thus, abundant adipose tissue produces leptin, a prosperity signal to the brain, and then the brain sends neuronal signals back to adipose tissue to increase energy expenditure. Importantly, there are two general types of adipose tissue in humans and other mammals. WAT stores chemical energy as triacylglycerols for use during periods of fasting or starvation. BAT also contains triacylglycerols but is enriched in mitochondria and adrenergic (sympathetic) innervation, both of which contribute to its brown color. BAT expresses uncoupling protein-1 (UCP1), which causes its mitochondria to dissipate the energy from oxidation of macronutrients as heat rather than harnessing this energy to make ATP. In human infants and in small rodents, which have high surface area-to-volume ratios, a major function of BAT is to maintain body temperature during cold exposure. Research during recent years provided unequivocal evidence for the existence of metabolically active BAT in normal human adults as well (24–26).

Leptin action in the hypothalamus, in part through local activation of the extracellular signal-regulated kinase (ERK) (27), increases sympathetic outflow to β_3_-adrenergic receptors on BAT adipocytes, thereby stimulating UCP1 expression and thermogenic activity (28). Several hypothalamic nuclei have been shown to be involved in activation of BAT and browning of WAT by leptin and other factors [reviewed in (29)]. Leptin acts in the mediobasal hypothalamus also to suppress WAT lipogenesis in an STAT3-independent manner (30). The fact that *db/db* mice (17) and leptin-deficient *ob/ob* mice (*Lep^ob/ob^*) (31) are cold intolerant supports a key role for leptin in heat production. Restoration of CNS expression of functional LepRb in *db/db* mice also restores cold tolerance (17). Abundance and activity of BAT or brown-like fat is reduced or absent in obese humans (25, 32), suggesting that energy expenditure could be substantially increased in these individuals if therapies could be found to stimulate BAT thermogenic activity and the browning of WAT (33). Owing to issues of safety and efficacy, however, no pharmacologic agents (34, 35) or devices (36) that were designed primarily to stimulate BAT have yet achieved regulatory approval for weight loss. Some data indicate pharmacological activation of the glucagon-like peptide-1 (GLP-1) system might increase BAT thermogenesis. For example, central administration of GLP-1 and GLP-1 receptor agonists has been shown to increase BAT thermogenesis in mice (37, 38).

Stimulation of WAT to increase their content of mitochondria and UCP1, a process called browning, has been shown to increase energy expenditure and suppress high-fat diet-induced obesity in rodents (39–41). Recently, Dodd and colleagues demonstrated that leptin acts on POMC neurons to promote the browning of WAT (42). WAT browning has been demonstrated in humans as well (43). Per gram of tissue, WAT after browning shows a lower thermogenic capacity compared with BAT (44). Nevertheless, there is much more WAT mass than BAT mass, suggesting that browning of WAT could substantially increase overall energy expenditure.

## Defective Central Responses to Leptin in Obesity

As noted earlier, obese individuals typically exhibit hyperleptinema, yet this overabundant leptin loses its normal appetite-suppressing effects (13–15). Strikingly, the ARC becomes selectively leptin resistant in mice with diet-induced obesity, whereas other hypothalamic and extrahypothalamic nuclei remain leptin responsive (15, 45). Also, there is evidence in diet-induced obese mice that exogenous leptin still activates BAT, even though leptin no longer suppresses food intake (45). Stellate cells in the liver also appear to retain their responsiveness to leptin in obesity; the hormone stimulates those cells to synthesize collagen and may thereby contribute to hepatic fibrosis and cirrhosis (46).

Several different mechanisms have been hypothesized to impair leptin responsiveness in the ARC in obesity [reviewed in Ref. (47)], including defective transit across the blood–brain barrier (BBB) that lines the ARC, hypothalamic endoplasmic reticulum (ER) stress, maladaptive sterile inflammation in the hypothalamus, and inhibited LepRb signaling owing to abnormal overexpression of suppressor of cytokine signaling 3 and protein tyrosine phosphatases (PTPases). Leptin transport across the BBB has been reported to be gradually impaired during high-fat feeding (48). In humans, the ratio of leptin concentrations in cerebrospinal fluid versus serum was found to be 4.3-fold higher in lean individuals than in obese individuals (49). Tanycytes, which are specialized glia in the BBB, have been reported to bring circulating leptin into the hypothalamus (50, 51), and there is evidence that leptin transport requires ERK activation in these cells (50). Recently, it was reported that ER stress in the ventromedial hypothalamus in obese Zucker rats leads to reduced BAT thermogenesis and weight gain, which could be rescued by overexpression of a chemical chaperone (52). Furthermore, it has been shown that histone deacetylase 5 activity is a regulator of leptin signaling (53). This picture is further complicated by evidence of discrepancies between endogenous and exogenous leptin sensitivity (54). Using a leptin receptor antagonist, Ottaway and colleagues concluded that diet-induced obese mice have essentially normal sensitivity to their endogenous leptin, despite other work indicating impaired sensitivity to exogenous leptin and the fact that these mice continue to overeat. Different animal models of obesity can give different results, and we recently reviewed problems with high-fat-fed rodent models of obesity (21).

Hypothalamic neurons and other cell types contain PTPases that dephosphorylate specific tyrosyl residues in canonical leptin and insulin signaling cascades, thereby attenuating or terminating the action of these hormones [Figure 1; Ref. (55)]. Accordingly, abnormal increases in the abundance (and possibly activity) of hypothalamic PTPases have been implicated in high-fat diet-induced obesity and central leptin resistance (56, 57). Conversely, genetic deletion of specific PTPases in the brain promotes leptin signaling in the ARC in association with decreased food intake, increased energy expenditure, and hence reduced adiposity. For example, deletion of protein tyrosine phosphatase 1B and T-cell protein tyrosine phosphatase enhances leptin signaling in POMC neurons and prevents diet-induced obesity by increasing WAT browning and energy expenditure (42).

Levels of PTPase activity in fasted, lean animals are sufficiently high to quickly undo the phosphorylation of key tyrosyl residues in LepRb, JAK2, and STAT3. Thus, for normal leptin signaling to propagate, hypothalamic neurons require a robust system to transiently inactivate PTPases that otherwise interfere with phosphorylation and activation of LepRb, JAK2, and STAT3. The enzymatic activity of PTPases depends on the presence of a reduced cysteine in a conserved motif, CX_5_R(S/T), within the active site (58, 59). Thus, certain members of the PTPase gene family are among the most redox-sensitive molecules in the cell. PTPase activity is regulated by reversible oxidation of that key active-site cysteine, and any disturbance in this process will affect leptin signaling.

We and others have shown that leptin normally induces a transient burst in reactive oxygen species (ROS) in neurons and other cell types (22, 60–62). Leptin seems to induce an increase in an ROS species with a long half-life—namely, hydrogen peroxide (H_2_O_2_; see below). Importantly, Diano et al. reported lower hypothalamic content of ROS in obesity and that central injection of low, non-toxic amounts of H_2_O_2_ mimicked the appetite-suppressing effects of leptin and restored leptin sensitivity in diet-induced obese mice (61). Nevertheless, the molecular mechanism by which leptin normally stimulates ROS production had remained uncharacterized.

## The Newly Discovered NSAPP Signaling Pathway is Essential for Canonical Leptin Signaling in Hypothalamic Neurons

Recently, our group discovered a new signaling pathway that we named “NSAPP” after its major protein components [red in Figure 1; (21, 23)]. The pathway consists of an oxide transport chain, in which certain hormones stimulate NADPH oxidase-4 (NOX4) to generate the superoxide ion $\left({\text{O}}_{2}^{\cdot -}\right)$. NOX4 hands ${\text{O}}_{2}^{\cdot -}$ to the nearby superoxide dismutase-3 (SOD3) for efficient conversion into H_2_O_2_. This H_2_O_2_ is generated extracellularly and requires aquaporins to cross the plasma membrane to enter the cell. Inside the cell, the H_2_O_2_ is targeted to inactivate redox-sensitive PTPases and the closely related enzyme PTEN. We initially showed that the NSAPP oxide transport chain is required for normal, balanced insulin signaling through the PI3K–AKT pathway in liver and in endothelium (21, 23). The NSAPP pathway fails to function normally in states of overnutrition, thereby providing a molecular explanation for pathway-selective insulin resistance, also known as imbalanced insulin action (21, 23).

In recent work (22), we found that all proteins of the NSAPP oxide transport chain are present in rat hypothalamus. In murine hypothalamic cell lines, leptin induced a burst in intracellular staining by a fluorogenic probe for ROS that we definitively identified as H_2_O_2_ by its quenching by catalase. Inhibition of NOX4 with diphenyliodonium abolished the leptin-induced H_2_O_2_ burst and blocked leptin signaling to key tyrosine phosphorylation sites on JAK2 and STAT3. Strikingly, knockdown of *Sod3* also blocked leptin signaling to JAK2 and STAT3. Consistent with our findings, NOX4-deficient mice are unusually susceptible to diet-induced obesity and early-onset insulin resistance for handling glucose (63). Moreover, the anorexigenic effect of insulin requires an increase in hypothalamic ROS mediated through NADPH oxidases that is blunted in high-fat diet-fed mice (64).

The NSAPP oxide transport chain finally provides a molecular explanation for how leptin normally provokes an ROS burst in hypothalamic neurons (22). Moreover, the NSAPP pathway is essential for canonical leptin signaling to JAK2 and then STAT3, which in turn regulates the expression of key neuropeptides, such as POMC and AgRP, that control appetite (Figure 1).

## Outlook

Remarkably, leptin and insulin both require the NSAPP oxide transport chain, suggesting that a defect in this pathway could explain simultaneous resistance to the appetite-suppressing effects of both hormones in obesity. Thus, we hypothesize that interference with the NSAPP signaling pathway in the hypothalami of lean animals will produce defects in central control of food intake and energy expenditure, causing overeating, positive caloric imbalance, and weight gain. In the other direction, unraveling the molecular basis for NSAPP dysfunction in overnutrition has now become a top priority. At this point, restoration of normal hypothalamic NSAPP function in obesity should be considered as an attractive, but entirely unexplored, strategy to promote weight loss.

## Author Contributions

All the authors reviewed the literature and approved the final version of manuscript. SF wrote the first draft. HV, AS, and KJW finalized the manuscript.

## Conflict of Interest Statement

KJW has an ownership interest in Hygieia, Inc., which provides insulin management services in Northern Ireland and in Michigan, USA. The other authors declare that they have no conflicts of interest. The reviewer RN and handling Editor declared their shared affiliation.
